# Horizontal gene transfer provides insights into the deep evolutionary history and biology of *Trichinella*

**DOI:** 10.1016/j.fawpar.2022.e00155

**Published:** 2022-04-18

**Authors:** Dante Zarlenga, Peter Thompson, Makedonka Mitreva, Bruce A. Rosa, Eric Hoberg

**Affiliations:** a U.S. Department of Agriculture, Agricultural Research Service, Animal Parasitic Diseases Laboratory, B1180 BARC-East Beltsville, MD 20705, USA; b Department of Medicine, Washington University School of Medicine, St. Louis, MO 63110, USA; c McDonnel Genome Institute, Washington University in St. Louis, St. Louis, MO 63108, USA; d Department of Biology and Museum of Southwestern Biology, University of New Mexico, Albuquerque, NM 87131, USA; e Department of Pathobiological Sciences, School of Veterinary Medicine, University of Wisconsin, Madison, WI 53706, USA

**Keywords:** *Trichinella*, Cyanase, Evolution, Horizontal gene transfer, Nurse cell

## Abstract

Evolution involves temporal changes in the characteristics of a species that are subsequently propagated or rejected through natural selection. In the case of parasites, host switching also plays a prominent role in the evolutionary process. These changes are rooted in genetic variation and gene flow where genes may be deleted, mutated (sequence), duplicated, rearranged and/or translocated and then transmitted through vertical gene transfer. However, the introduction of new genes is not driven only by Mendelian inheritance and mutation but also by the introduction of DNA from outside a lineage in the form of horizontal gene transfer between donor and recipient organisms. Once introduced and integrated into the biology of the recipient, vertical inheritance then perpetuates the newly acquired genetic factor, where further functionality may involve co-option of what has become a pre-existing physiological capacity. Upon sequencing the *Trichinella spiralis* (Clade I) genome, a cyanate hydratase (cyanase) gene was identified that is common among bacteria, fungi, and plants, but rarely observed among other eukaryotes. The sequence of the *Trichinella* cyanase gene clusters with those derived from the Kingdom Plantae in contrast to the genes found in some Clade III and IV nematodes that cluster with cyanases of bacterial origin. Phylogenetic analyses suggest that the *Trichinella* cyanase was acquired during the Devonian period and independently from those of other nematodes. These data may help inform us of the deep evolutionary history and ecological connectivity of early ancestors within the lineage of contemporary *Trichinella*. Further, in many extant organisms, cyanate detoxification has been largely superseded by energy requirements for metabolism. Thus, deciphering the function of *Trichinella* cyanase may provide new avenues for treatment and control.

## 1 Introduction

Horizontal or lateral gene transfer (HGT) involves the conveyance of genetic material between donor and recipient organisms even across kingdoms, by other than vertical or sexual inheritance. The biological mechanisms that direct such a transfer have not been elucidated. As with all traits persisting as evolutionarily conserved attributes, those acquired by HGT are assumed to impart selective advantage and be important in the biological character of the recipient organism (Blaxter, 2007). If this requirement is not met, the gene risks becoming a nonfunctional relic or pseudogene, or one that is eventually lost in the process of natural selection. For this reason, HGT involves the acquisition of genes encoding traits that are beneficial to the recipient organism. Some have proposed that orthologous replacement of existing genes may also occur where homologues present in the recipient's food source, symbionts or pathogens that impart greater fitness to the recipient can also be acquired (Doolittle et al., 2003); however, this can be a challenge to validate.

Once considered an anomaly, HGT has emerged as a prominent mechanism and outcome in prokaryotic evolution (Polz et al., 2013), but has remained enigmatic in the evolution of higher eukaryotes until more recently. Dropkin (1963) demonstrated the presence of cellulase activity in plant parasitic cyst nematodes years ago, but it was not until 1998 that Smant et al. (1998) discovered the presence of four functional cellulase genes in the cyst nematodes *Globodera rostochiensis* and *Heterodera glycines* that are typically found in soil bacteria. The encoded proteins help in the degradation of cellulose and xyloglucan which are key components of plants. Given the sequence similarity between these genes and those present in soil bacteria, Smant et al. (1998) suggested that these endoglucanases play a role in modifying plant cells into multinucleated feeding cells, or plant nurse cells (NC), for the nematodes and target all major cell wall carbohydrates to facilitate intercellular migration of the nematode in the host plant. They further concluded that in contrast to most omnivores and herbivores living symbiotically with cellulolytic microorganisms to facilitate digestion of cellulose, these plant parasitic nematodes acquired their genes through HGT from bacteria. Keen and Roberts (1998) simplified the data interpretation by suggesting that the cyst nematodes ‘borrowed’ the cellulase genes from bacteria that typically populated their digestive tracts and ultimately converted a once symbiotic relationship with plants into a parasitic lifestyle. The role of HGT in plant parasitic nematodes was further corroborated using comparative genomics (Mitreva et al., 2009). Haegeman et al. (2011) later suggested that gene transfer may have been a fundamental condition associated with the acquisition of parasitism in cyst nematodes given that HGT is widespread among independent lineages. The autonomous acquisition of a cyanase gene among a handful of parasitic nematodes that have at least one tissue dwelling stage maybe a link between HGT, parasitism and co-option of these metabolic pathways among vertebrate parasites (Zarlenga et al., 2019).

Andersson (2005) proposed multiple elements that are needed to validate that a gene is derived from HGT. Understandably, two of these elements encompass extensive phylogenetic analyses and exploration of patterns for gene distribution using both closely and more distantly related organisms. A third factor postulates that the gene must have become an integral member of the genetic repertoire and a biological character of the recipient organism (Blaxter, 2007). Finally, there must be some demonstration of a physical association between the recipient organism and its donor counterpart. Schliep et al. (2011) showed that among prokaryotes, the lifestyles of the organisms and occurrence in similar environments with the assumption of shared ecology, are more important in HGT than is taxonomy. This observation is consistent with use of a widespread resource by taxonomically divergent assemblages of organisms under common ecological circumstances. Such is the basis for ecological fitting in sloppy fitness space. This also relates to opportunity and organismal capacity across environmental interfaces and provides the ecological context to exploit a common (often trophic) resource, rather than adaption (Agosta et al., 2010; Agosta and Brooks, 2020). This last element is elusive and challenging to validate given that much of gene transfer likely occurred in early evolutionary history, and under ecological circumstances that are substantially different relative to conditions encountered among contemporary taxa. Thus, trophic associations of free-living ancestors (recipient organisms) may be substantially different than those associated with a parasitic life history, reflecting divergent selection pressures over time.

HGT is not supported among organisms that do not or did not at one time closely share a common ecosystem and/or symbiotic relationship including exploitation of conserved and widespread resources. Sequence similarity and phylogenetic reconstruction of the putative transferred gene provides a context for recognizing the history of interaction(s) and assimilation(s) between donor and recipient organisms. The necromenic, free-living nematodes in the genus *Pristionchus* are examples of the acquisition of genes through a shared ecological interface and may also represent the transitioning of *Pristionchus* spp. from a free-living to a parasitic lifestyle where numerous biological traits have been identified that are considered pre-adaptive for parasitism (Weller et al., 2010). Currently, *Pristionchus* spp. exhibit apparently species-specific beetle preferences (see Brooks et al., 2019 for discussion of specificity; Hoberg and Zarlenga, 2016), and associate with, but do not infect the beetles. Once the larvae attach to the beetle, worm development arrests in response to secreted beetle sex hormones. Worm development resumes only after the beetle dies and hormone secretion has ended. The nematodes then feed off the bacteria, fungi, and protozoa on the surface of the beetle. In one study, which was predicated on similarities among *Pristionchus* spp. and insect codon usage, computational archaeology identified over 500 *Pristionchus* genes that harbor insect-like characteristics (Rödelsperger and Sommer, 2011). This work followed the identification of multiple diapausin genes of putative insect origin, and cellulase genes of bacterial, fungal or plant origins within the *Pristionchus pacificus* genome (Dieterich et al., 2008; Mayer et al., 2011). Both the diapausin and cellulase genes are presumed to have been acquired by HGT and both have become integral elements of the biology of *Pristionchus* spp.

Through comparative genome analyses, we previously identified a cyanate hydratase (cyanase) gene in the Clade I (Blaxter et al., 1998; Meldal et al., 2007) parasitic nematode, *Trichinella spiralis* (Mitreva et al., 2011; Zarlenga et al., 2016; Zarlenga et al., 2019). Cyanase degrades toxic cyanate to ammonia and carbon dioxide. This gene clusters with a clade of cyanase genes found in plants and eukaryotic algae, in contrast to those present in bacteria or fungi and a restricted or narrow group of nematode species (Zarlenga et al., 2019; Mao et al., 2021). The prima facie data suggests independent events of HGT, and subsequent genomic incorporation are the basis for divergent cyanase pathways among nematodes. Given the dearth of information available on the evolutionary history of the Trichinellidae and the Trichuridae since they last shared a common ancestor 265–280 MYBP (Zarlenga et al., 2006; Korhonen et al., 2016), and knowing the obligate link among extant *Trichinella* and Eutherian mammals, it is challenging to envision a past assimilation between the Trichinellidae and members of the Kingdom Plantae. Further, it is equally challenging to rationalize the presence of a functional *Trichinella* cyanase gene in members of this genus through other than HGT. Based upon our data and those of others, we hypothesize that any putative association among these groups took place before the divergence of the Trichinellida-lineage during the Devonian. Data and analyses are consistent in postulating an association between members of the Kingdom Plantae or their symbionts, and early common ancestors among the Dorylaimea that gave rise to the Orders Trichinellida and Dioctophymatida. Essential questions that arise from this finding are: 1) How did such an acquisition take place? 2) Does it predate or coincide with parasitism in this lineage? 3) Was acquisition of the cyanase pathway phylogenetically widespread among basal Clade I nematodes then secondarily in most lineages but maintained among the assemblage of Trichinellida? 4) Did cyanase play a role (or not) in parasitism, early worm development and parasite-host relationships? and finally 5) What contemporary function does the enzyme play in parasite maintenance during a progression of life history stages. The concepts that follow are speculative in nature but nonetheless can generate testable hypotheses for future studies that relate to the involvement and importance of cyanase in the biology of *Trichinella.*

## 2 Phylogenetic analyses and distribution patterns of cyanase

Phylogenetic inference suggests a deep history for HGT in the ancestor of Dorylaimea, with retention of a cyanase pathway among the common ancestor of Trichocephalia which contains the obligate vertebrate parasites in Trichinelloidea (*Trichinella*, *Trichuris* among 6 families of zooparasitic nematodes) and Dioctophymatoidea (*Soboliphyme* and *Dioctophyme* among 2 families of zooparasites) along with an additional superfamily of parasites (Muspiceoidea among chiropterans and insects). HGT is unlikely in the common ancestor of these vertebrate parasites and appears consistent with a considerably older and different ecological regime during the time frames for transfer and genomic assimilation, although cyanases have yet to be demonstrated among other Dorylaimea. Incorporation of the cyanase pathway is postulated to have occurred across an ecological assemblage, and a diversity of apparently phytophagous free-living terrestrial nematodes, that are now among the most ubiquitous taxa in terrestrial and freshwater habitats. In contrast, the ancestor of Trichocephalia was already established in a parasitic life history pattern near 300 MYBP (e.g., Zarlenga et al., 2006) and as a consequence, the ecological conditions for acquisition of a plant-related metabolic pathway would not have been a factor.

HGT is consistent and analogous to processes associated with colonization events among parasites and hosts in the context of ecological fitting in sloppy fitness space (Agosta et al., 2010; Agosta and Brooks, 2020; Brooks et al., 2019; Hoberg and Brooks, 2008). Here, circumstances at environmental interfaces drive associations through the interaction of capacity (to exploit resources) and opportunity that provides access across taxa in space and time. Thus, the circumstances for HGT and initial incorporation in the genome are processes that are distinct from the downstream evolutionary regimes and selection that can modify newly acquired physiological capacity. How cyanases may have acted in early evolutionary associations among basal Dorylaimea can be fundamentally different than processes in contemporary time and within the crown Trichocephalia (superfamilies Dioctophymatoidea and Trichinelloidea which respectively contain *Soboliphyme* and *Trichinella + Trichuris*). Secondary modifications through selection in these divergent lineages may constitute trajectories for co-option in differing ecological and functional regimes associated with independent origins of parasitism (see Agosta and Brooks, 2020 for discussion of co-option). Persistence of cyanase pathways, restricted among a limited assemblage of nematodes, may represent genetic linkage or may contribute functionally to metabolism among this extant assemblage of genera and species.

**Fig. 1:**
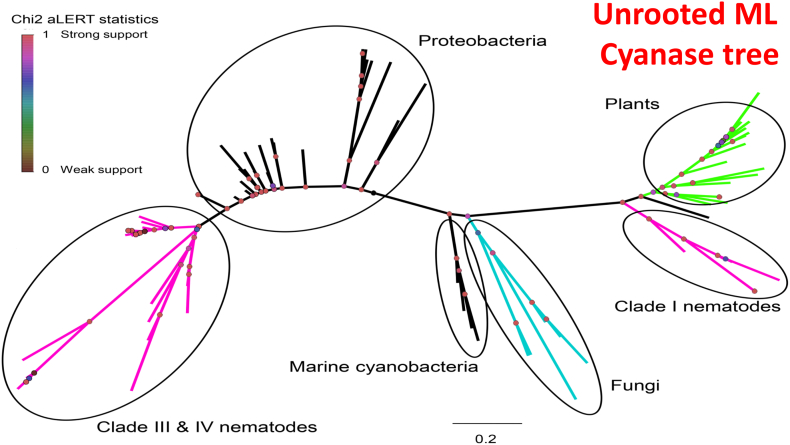
Unrooted maximum likelihood tree of cyanases. Lineages are color coded and include parasitic nematodes, bacteria, fungi, and plants. The tree shows distinction in the placement of Clade I nematodes (sister to plant cyanases) and those from Clades III and IV (cluster with bacterial cyanases). (Reprinted from Zarlenga et al., 2019).

In 2016, Zarlenga et al. (2016) examined *T. spiralis* in the context of the acquisition of or adaptation to parasitism. The data were consistent with a large loss in genes among the Nematoda but the independent gain in genes putatively associated with parasitism. The authors first noted the presence of a cyanase gene among a handful of nematodes and proposed that these genes were obtained independently through HGT and likely represented distinct evolutionary events in space and time. Work had not yet progressed to explore whether the acquired genes were functional, or merely evolutionarily conserved, widespread relics of past associations. A more extensive phylogenetic study ensued that encompassed 87 cyanase genes from three different Kingdoms, among which were 33 nematode sequences (Zarlenga et al., 2019). The results of a mid-point rooted phylogenetic tree are shown in Fig. 1. and are consistent with independent acquisition of cyanase, respectively, among Clade I and among Clade III + Clade IV Nematoda. The analysis further corroborated that the cyanase of Clade I nematodes, represented by *Trichinella*, *Trichuris,* and *Soboliphyme,* clustered with members of the Kingdom Plantae (with greater similarity to green algae which appeared within the plant group). All other nematode cyanases distributed among Clade III and Clade IV parasitic taxa and clustered with those derived from proteobacteria. Monophyly is not demonstrated by cyanases relative to taxon clearly indicating historically independent acquisition and pathways during radiation of the Nematoda. This was a rather startling discovery since in the contemporary fauna, species of these three genera of Trichinellida and Dioctophymida, among a considerable diversity of basal Dorylaimea (historically among Blaxter's Clade I), are obligate mammalian parasites. Those analyzed among Rhabditida (Clade III) nematodes (Ascaridoidea- species of *Ascaris*, *Toxascaris* and *Anisakis*; Filarioidea- species of *Brugia*, *Loa* and *Onchocerca*) also circulate in mammalian definitive hosts. Those of the Clade IV Rhabditida (*Strongyloides*) and Tylenchida (*Meloidogyne*) include vertebrate and plant parasites with free-living stages, respectively.

**Fig. 2:**
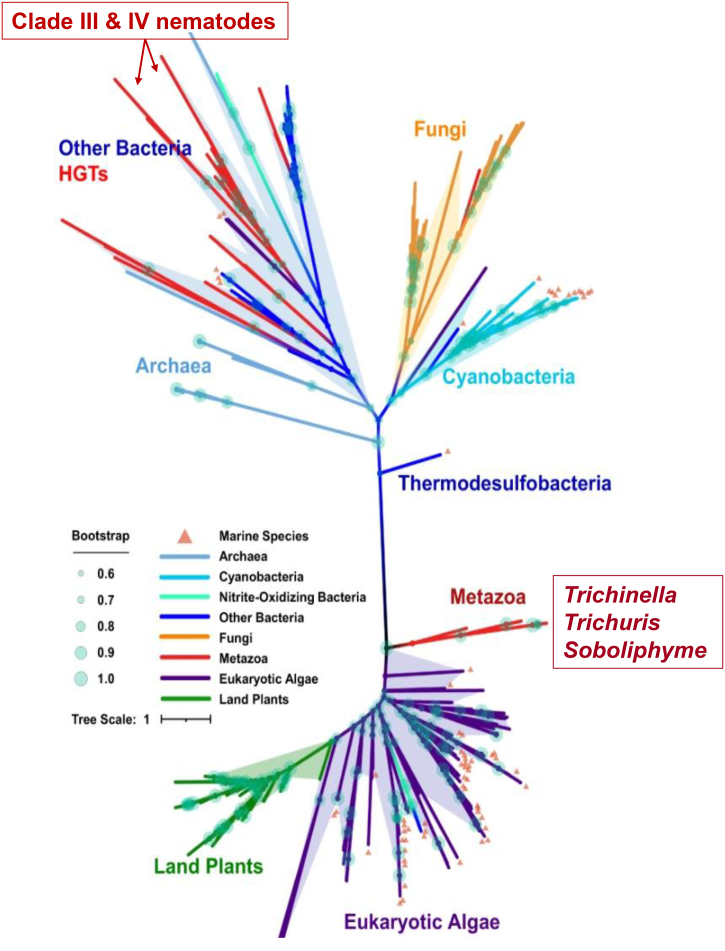
Unrooted maximum likelihood tree of cyanases. Key lineages are color-coded and include archaea, bacteria, metazoan, fungi, eukaryotic algae, and land plants. Distinctions among the parasitic nematodes are presented. (Reprinted from Mao et al., 2021 with modification).

More recently, Mao et al. (2021) examined cyanase genes from 260 organisms that span the Tree of Life (Fig. 2) including nematode sequences. Their analysis provided a more extensive representation of bacteria including thermodesulfobacteria, along with a more robust collection of marine-based, eukaryotic algae and land plants. Like the data presented by Zarlenga et al. (2019), the cyanases found in nematodes were partitioned among bacterial and plant sources. Clade III and IV nematodes likely acquired the cyanase in one or more independent events from bacteria whereas one group defined as “Metazoan” in Fig. 2 included nematodes of Clade I, species of *Trichinella*, *Trichuris* and *Soboliphyme* which consistently clustered with plant cyanases.

**Supplementary Fig. S1:**
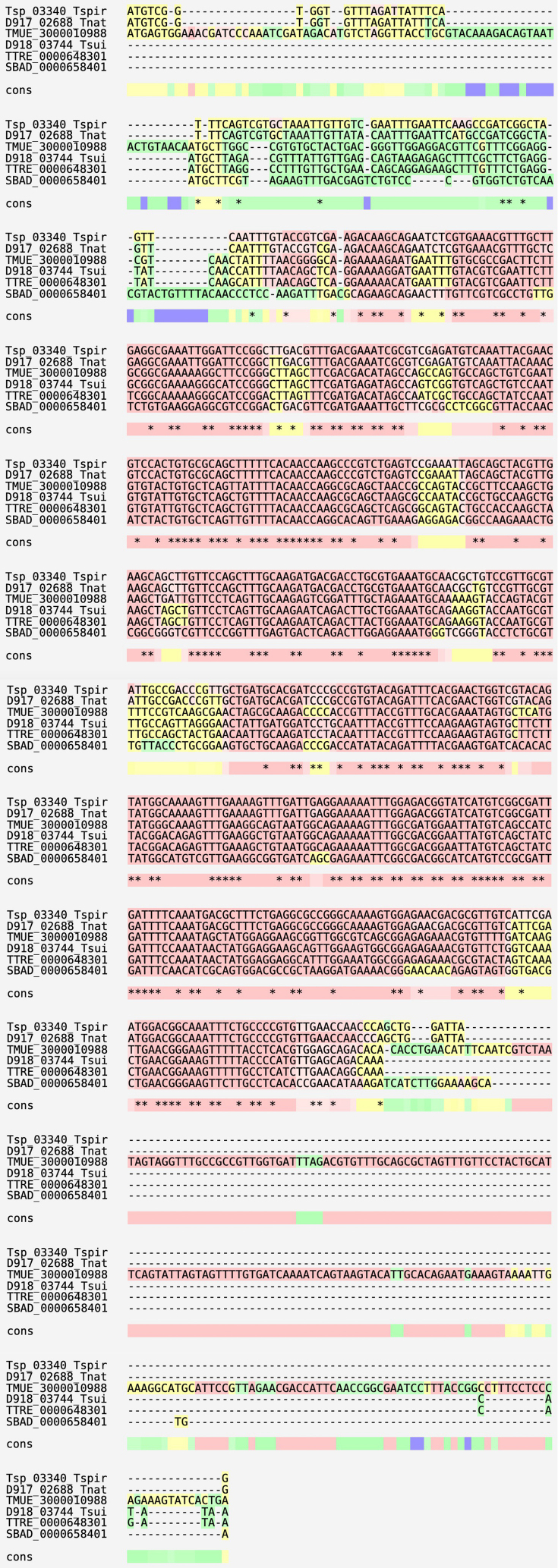
Multiple sequence alignment of cyanase genes. Genomic sequence alignments for the cyanase genes appear in order as *Trichinella spiralis*, *Trichinella nativa*, *Trichuris muris*, *Trichuris suis*, *Trichuris trichiura*, and *Soboliphyme baturini*. Alignment was performed with M-COFFEE (part of the T-COFFEE package, version 11.00). Color coding indicates the level of agreement between eight aligners combined for the final alignment (blue = very poor, green = poor, yellow = average, pink = good, red = perfect).

Upon aligning genomic sequences from *Trichinella*, *Trichuris* and *Soboliphyme*, we found that the cyanase genes exist as single exons except for *T. muris*; however*,* this is likely attributable to improper sequence annotation (Supp Fig. 1). Among all the genes that comprise the *T. spiralis* genome, approximately 14% are single exons so the absence of introns is not uncommon in this genus. Cyanase genes from a subset of Clade IIIa nematodes including *Ascaris lumbricoides, Toxocara canis* and *Anisakis simple*x, each contain a single intron; members of Clade IIIc *Brugia malayi*, *Onchocerca volvulus* and *Loa loa* possess two introns; and *Strongyloides stercoralis* (clade IVa) and *Meloidogyne hapla* (Clade IVb) possess one and three introns, respectively (for Clade designations see International Helminth Genomes Consortium, 2019). Since these genes are believed to have been derived from bacterial sources, the presence or absence of introns is not a reliable indicator of the species of origin of the gene. Also, a cursory evaluation of the sequence databases finds that some eukaryotic algae possess cyanase genes that do not harbor introns.

Previously, Pachiadaki et al. (2017) and Widner et al. (2018) demonstrated that cyanases from the nitrifying bacteria *Nitrospina* did not cluster with bacterial cyanases but were more similar to those from eukaryotic algae. This was more recently corroborated by Zarlenga et al. (2019) and Mao et al. (2021). However, this character is not shared by other nitrifying bacteria such as *Nitrospira.* Consequently, Ushiki et al. (2018) hypothesized that HGT played a role in cyanase acquisition even among nitrifying bacteria*.* Widner et al. (2018) further noted that cyanases of the phylum Planctomycetes (genus *Scalindua*) which are marine, ammonium-oxidizing organisms also clustered with the Nitrospinae embedded within the eukaryotic algae lineages rather than in a separate, proximal clade. The authors concluded that this was likely the result of HGT among the eukaryotic algae, *Nitrospina* and *Scalindua,* which often co-exist in nature. One might not expect such diversity among nitrifying bacteria; however, extant *Nitrospira* and *Nitrospina* exist within distinct ecologies and commonly inhabit eutrophic (high nutrient values) and oligotrophic (low nutrient values) marine locations, respectively (Nunoura et al., 2015). Also, epiphytic Planctomycetes communities to which *Scalindua* spp. belong, frequently exist as biofilms on macro- and microalgae, sponges and lichens and thus live in association with specific eukaryotes which harbor similar cyanase genes (Bondoso et al., 2017; Kaboré et al., 2020; Wahl et al., 2012). If extant or extinct eukaryotic algae-associated bacteria were the source of cyanase in the Clade I nematodes, their existence predominantly in marine environments may help define a timeline for gene acquisition. It should be noted that Archibald et al. (2003) performed extensive work on HGT in algae. They showed that conservatively, 21% of genes in the eukaryotic amoeboflagellate algae *Bigelowiella natans* that encode plastid proteins, were likely acquired via HGT from a multitude of exogenic sources including bacteria, other algae, and algal endosymbionts. Primary and secondary endosymbiosis have been proposed as key factors in the acquisition and movement of genes among many forms of algae. Thus, HGT has been recognized as instrumental in shaping the evolution of eukaryotic algae.

**Fig. 3:**
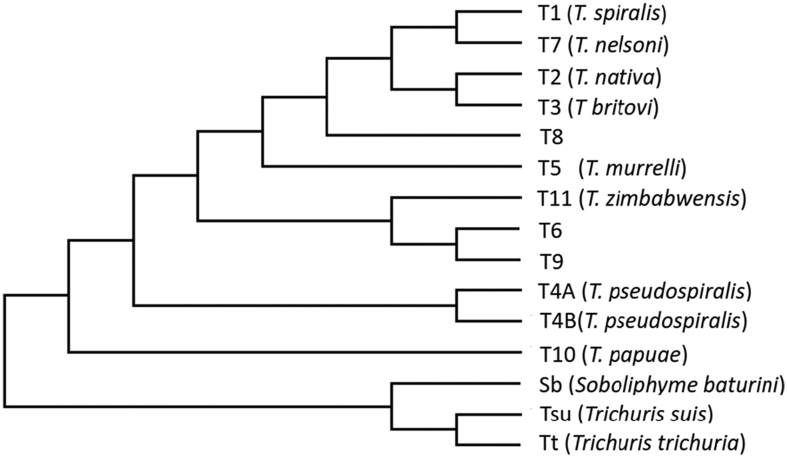
Unrooted, maximum likelihood tree of *Trichinella* cyanases including those from *Trichuris* spp. and S*oboliphyme*. Alignment was performed with CLUSTAL (part of the MEGA-11 package). Gaps were removed at the variable N-terminus prior to tree building. Phylogenetic classifications for *Trichinella* genotypes T6, T8 and T9 remain unresolved. Protein accession numbers and DNA reference numbers (*Soboliphyme*) for the aligned sequences are as follows: T1 = EFV60696; T2 = OUC42830; T3 = QTG10995; T4A = KRX89740; T4B = ABR10530; T5 = KRX40590; T6 = KRX81296; T7 = KRX22994; T8 = KRZ91604; T9 = KRX64400; T10 = KRZ67954; T11 = ABR10534; Tsu = KHJ46081; Tt = CDW58178; Sb = SBAD_0000658401.

Higher plants exhibit a unique character where they can generate mobile mRNAs that are translocated among cells and parasitic plant species. The activity is mediated locally by non-cell-autonomous proteins (Lucas et al., 2001) and may help coordinate plant development via RNA-based signaling (Roney et al., 2007). Others have shown that plant plasmodesmata which is important in the trafficking of macromolecules in plants is also involved in translocating processed mRNAs (Oparka and Turgeon, 1999). Roney et al. (2007) proposed this function as an alternative method for interspecies communication and suggested that the mobile mRNAs may provide a mechanism for parasitic plants to identify and use host transcripts. Although the majority of HGT genes in plants derive from genomic DNA (Kado and Innan, 2018), mobile RNAs which lack introns have been proposed as a mechanism for HGT (Yoshida et al., 2010). Based on low coverage sequencing of extant species of *Trichinella* (Korhonen et al., 2016), sequence data for cyanase genes were extracted from all but the most recently proposed nominal species, *Trichinella patagoniensis* and *Trichinella chanchalensis*. Phylogenetic reconstruction using the full-length sequences is shown in Fig. 3 and includes data from *Trichuris,* and *Soboliphyme.* As expected, the non-encapsulated *Trichinella* species clustered independent of the encapsulated species except for *T*. *zimbabwensis* (T11) which formed a clade with genotypes T6 and T9. It is likely that small differences in the amino acid sequence accounted for the lack of congruence between the cyanase tree and other accepted phylogenies of *Trichinella* (Korhonen et al., 2016; Zarlenga et al., 2006); however, the data are consistent with the gene having remained under evolutionary pressure, where the active site exhibits but one amino acid change that differentiates the encapsulated from non-encapsulated clades. Cyanase genes have yet to be identified in free-living nematodes or in more recently diverged nematode taxa i.e., those belonging to Clade V as defined by Blaxter et al. (1998); however, further evidence of independent evolutionary trajectories and cyanase acquisition is exemplified among ectoparasitic mites (Wybouw et al., 2012) suggesting a link between HGT, cyanase and parasitism.

## 3 Activity and operational functionality of the Trichinella cyanase

**Fig. 4:**
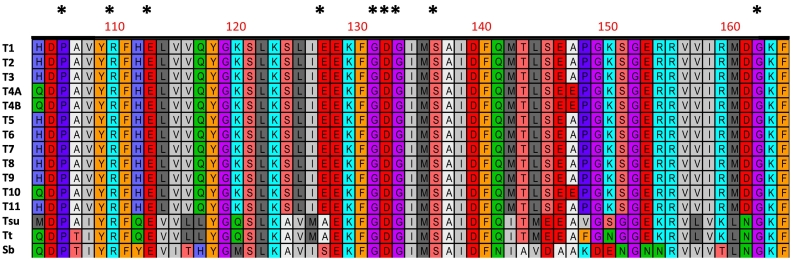
Alignment of cyanase active sites for species and genotypes of *Trichinella*. Amino acids designated with “*” indicate key loci for enzyme activity as per Mao et al., 2021. The species and genotype designations are defined in the legend to Fig. 3 as are the accession numbers from which these sequences were obtained and the alignment parameters.

We assume that persistence (evolutionary conservation) of a gene demonstrated to have origins related to HGT must have an essential physiological role. The first step in determining if a gene is important to the organism is to ascertain if the gene product is in fact operational. To this end, Zarlenga et al. (2019) demonstrated that a recombinant *T. spiralis* cyanase was capable of degrading cyanate to ammonia in the presence of bicarbonate, and that the activity could be curtailed by sodium chloride, a known competitive inhibitor of cyanases (Anderson and Little, 1986; Anderson et al., 1987). Immunohistochemical staining revealed the presence of the cyanase in the hypodermis and in the adjoining muscles of the L1, as well as the glandular cell nuclei in the stichosome. If the muscle larvae (ML) within the NC acquire nutrients via movement across the cuticle, this location would be the first line of defense against cyanate. Alignment of the active site of *T. spiralis* cyanase showed that the cyanases from *Trichinella* spp. as well as *Trichuris,* and *Soboliphyme* were conserved at 9 canonical loci that define cyanase activity (Mao et al., 2021) suggesting that all the genes encode proteins that are functionally active (Fig. 4).

**Fig. 5:**
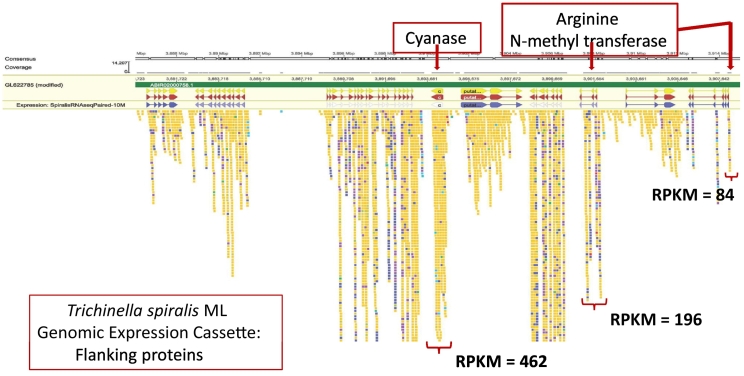
Genome expression cassette for *T. spiralis* cyanase. The locations for the cyanase gene and the two arginine *N*-methyl transferase genes are defined. Transcript read coverage is represented by the colored blocks, and normalized expression levels (RPKM = reads/kilobase of gene length/10^6^ mapped sequence reads) are indicated for the genes of interest.

**Supplementary Fig. S2:**
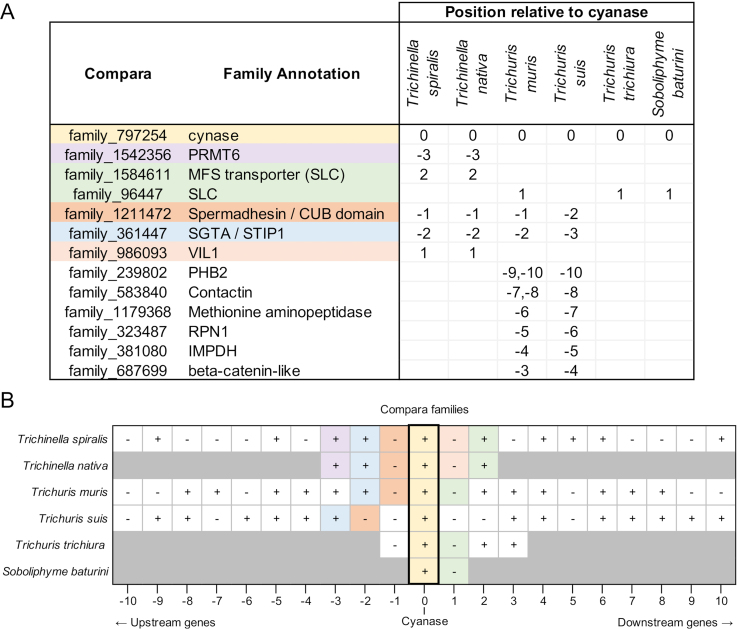
The positions of genes assigned to different Compara families relative to the cyanase gene in species of interest. (A) Compara family membership (identified in IHGC, 2019) among the 10 genes upstream and downstream of cyanase in each of the species of interest. Only families identified in two or more species are shown. Numbers indicate the position of the genes relative to cyanase (0 = cyanase, negative = upstream, positive = downstream). Color coding is used to indicate functions of interest. (B) Positional orientation of genes upstream and downstream of cyanase in species of interest. Color coding corresponds to the colors shown in panel (A), and greyed-out genes indicate that no other genes are present further upstream/downstream on the contig containing the cyanase gene. Plus, signs indicate genes oriented on the same strand as cyanase, and minus signs indicate genes on the opposite strand as cyanase.

**Supplementary Fig. S3:**
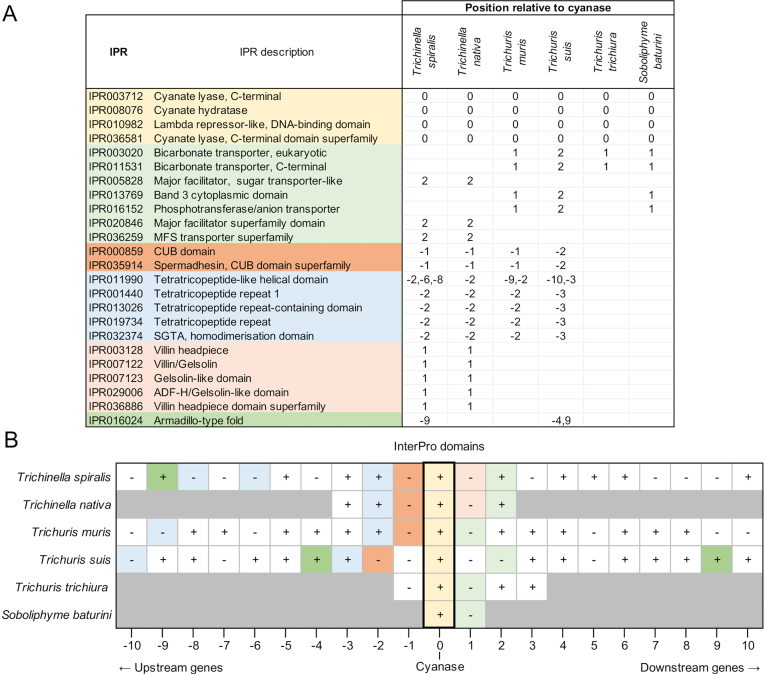
The positions of genes annotated with different InterPro domains, relative to the cyanase gene in species of interest. (A) InterPro (IPR) domain annotations (identified in IHGC, 2019) among the 10 genes upstream and downstream of cyanase in each of the species of interest. Only IPR domains identified in two or more species are shown. Numbers indicate the position of the genes relative to cyanase (0 = cyanase, negative = upstream, positive = downstream). Color coding is used to differentiate overall biological functions of interest. (B) Positional orientation of genes upstream and downstream of cyanase in species of interest. Color coding corresponds to the colors shown in panel (A), and greyed-out genes indicate that no other genes are present further upstream / downstream on the contig containing the cyanase gene. Plus, signs indicate genes oriented on the same strand as cyanase, and minus signs indicate genes on the opposite strand as cyanase.

**Supplementary Fig. S4:**
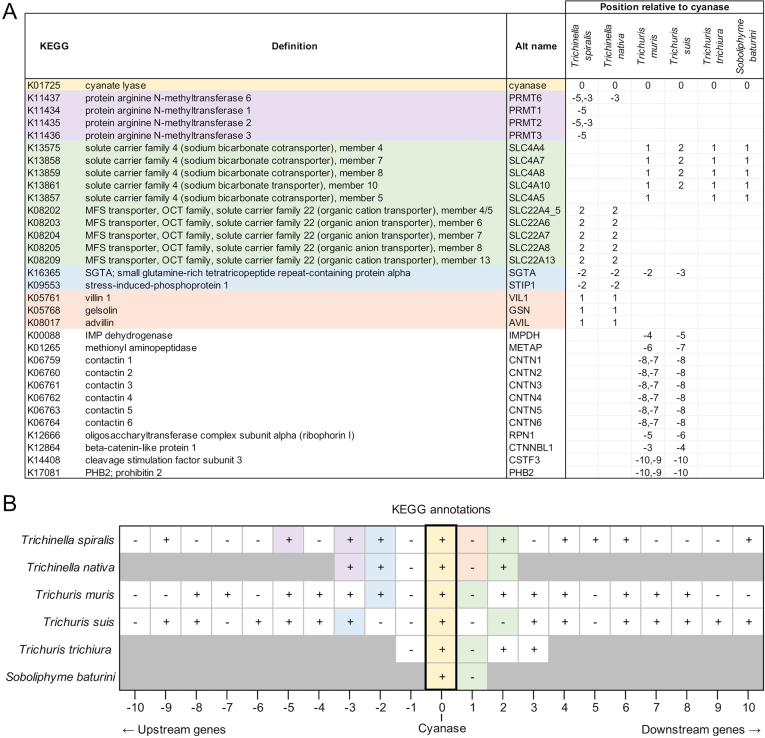
The positions of genes annotated as different KEGG proteins, relative to the cyanase gene in species of interest. (A) KEGG annotations (identified in IHGC, 2019) among the 10 genes upstream and downstream of cyanase in each of the species of interest. Only KEGG annotations identified in two or more species are shown. Numbers indicate the position of the genes relative to cyanase (0 = cyanase, negative = upstream, positive = downstream). Color coding is used to differentiate overall biological functions of interest. (B) Positional orientation of genes upstream and downstream of cyanase in species of interest. Color coding corresponds to the colors shown in panel (A), and greyed-out genes indicate that no other genes are present further upstream/downstream on the contig containing the cyanase gene. Plus, signs indicate genes oriented on the same strand as cyanase, and minus signs indicate genes on the opposite strand as cyanase.

We examined synteny proximal to the cyanase *(cyn)* gene in *Trichinella* spp. *Trichuris* spp. and *Soboliphyme.* Annotations were collected based upon Compara orthologous protein family membership and KEGG data from the International Helminth Genomes Consortium (2019), and InterPro domains and Gene Ontology from WormBase Parasite (Howe et al., 2016, Howe et al., 2017). Results showed substantial overlap in *Trichinella* and *Trichuris* genes where the databases are more robust (Supp Fig. 2). Less similarity was observed when data from *Soboliphyme* was included; however, this likely resulted either from limited sequence information or from deviations in evolutionary trajectories. When examining Interpro domains shared by two or more species (Supp Fig. 3), all species with two or more genes upstream of cyanase contained a CUB domain/Spermadhesin gene followed by an SGTA/Tetratricopeptide gene. The CUB domain/Spermadhesin gene is developmentally regulated and, among other things, is involved in tissue repair and angiogenesis; two activities engaged by a recovering NC. In the KEGG analysis, all organisms possessed an SLC transporter adjacent to or proximal to the cyanase gene. Many SLC transporters are responsible for arginine transport (Dall'Asta et al., 2000; Fang et al., 2007; Tsumuraya and Matsushita, 2014) which may be the function in *Trichinella* since these are solute carriers (Supp Fig. 4). Also, within the *Trichinella* cassette there exists one or more genes encoding protein arginine *N*-methytransferase (Fig. 5) which regulates protein activity via arginine methylation. Although posttranslational methylation of arginine is known to regulate signaling pathways related to cell differentiation and proliferation by inhibiting or activating transcription factors and other proteins, arginine is at the core of the cyanase active site and is a member of the canonical amino acids conveying activity potentially linking the arginine *N*-methytransferase to cyanase activity. In contrast, key transporters associated with the cyanase gene in *Trichuris* and *Soboliphyme* species were of the sodium bicarbonate type. In the catabolism of cyanate to ammonia and CO_2_, both bicarbonate and cyanate function as substrates, but bicarbonate acts as a recycling substrate (Johnson and Anderson, 1987). These data suggest that this part of the cassette may have been acquired during HGT as well, but trophic changes led to the loss or replacement of this gene in *Trichinella* which harbors a small solute transporter. A common thread nonetheless with the cyanase cassette in bacteria is the presence of a solute transporter which may be important in arginine transport.

Following the genomic incorporation of a cyanase pathway in ancestral Dorylaimea, it appears to have been conserved among the Trichocephalia. Thus, it is the ecological context of the ancestor prior to the “radiation” that explains the conditions and the opportunity for HGT. Following HGT it may have been retained by genetic linkage or co-opted given that the ecology of these organisms indeed changed since HGT took place. A process of co-option cannot occur until the gene and the pathway have first been incorporated in the genome. In this, the explanation resides in the idea of an evolutionarily conserved pathway (what we call a pre-existing capacity) that has been co-opted (how and for what in the context of selection, not adaptation). Further it is likely that this is evolutionary baggage once served a different function in the ancestral lineage but under a different set of ecological circumstances, and with the transition to parasitism during nematode radiation, primary functionality likely shifted. Today we see the outcome of an independent shift in physiological capacity in deep time that has become conserved over the scope of evolutionary time. The history for acquisition and persistence of cyanase and its current link to *Trichinella* physiology may be advanced by looking at the function of cyanase in other systems. In general, these activities can be narrowed down to detoxification and cyanate assimilation as a source of energy. Thus, we can explore how mechanisms have been co-opted and modified following HGT.

### 3.1 Cytoplasmic detoxification of cyanate

The challenge of HGT is integrating biochemical activity and physiology with the proviso that metabolic pathways and other attributes can become evolutionarily conserved and later co-opted reflecting different drivers between initial acquisition and the later history of selection. Dieterich and Sommer (2009) proposed, with examples from the necromenic nematode, *P. pacificus,* that the acquisition of detoxifying enzymes must occur during pre-adaptation (Osche, 1956) [more correctly co-option] when a free-living organism begins transitioning to a new or host environment. Co-option is the process where conserved attributes are “re-purposed” for a secondary or novel function and represent the use of available capacity within the genome to respond to dynamic and changing conditions (Agosta and Brooks, 2020; Brooks et al., 2019). Also, co-option reflects outcomes from deep histories of phylogenetic conservatism and should not be confused with “preadaptation” which is not a process in the sense implied by Osche (1956). Could this theory explain how ancestors among the Dorylaimea acquired the cyanase gene, and how through co-option it is now manifested among Trichinellida? A look to the chemistry of primordial earth might provide some clues.

Whether it be chemistry or cosmic intervention, most agree that prebiotic earth existed in a weakly reducing atmosphere dominated by N_2,_ CO_2,_ CH_4_ and H_2_O (for review see Cleaves, 2012). The generation of hydrogen cyanide (HCN) from this mire is believed to have been instrumental in the development of life on earth (Das et al., 2019; Ghoshal et al., 2020) and has been linked to the early synthesis of purines and amino acids (for review see Trainer, 2013). Further, the destruction of HCN is dependent upon the carbon/oxygen ratio where atomic oxygen will break down HCN to cyanate, albeit a short-lived molecule i.e., HCN + O → NCO + H (Rimmer and Rugheimer, 2019). Aqueous cyanate has been linked to the prebiotic synthesis of pyrimidines via an interaction with cyanoacetylene and a cyanovinylurea intermediate (Ferris et al., 1968). Further, Yamagata (1999) showed that in the presence of calcium phosphate, cyanate could have assisted in the production of ATP. With the beginning of the Great Oxidation Event (GOE) over 2.5 billion years ago (Holland, 2002, Holland, 2006), environmental cyanate likely increased from oxygen generating cyanobacteria (Tomitani et al., 2006) which flourished during his period. Additionally, ammonium cyanate can be generated from the decomposition of urea (Dirnhuber and Schütz, 1948) where urea is also considered a key intermediate in prebiotic earth and a potential precursor of nitrogenous heterocycles and therefore nucleobases. (Menor Salván et al., 2020; Nowakowska et al., 2006). Albeit toxic to cells, the importance of cyanate in earth's development and its availability as a timely biochemical substrate cannot be overstated.

Many early forms of bacteria such as cyanobacteria, synthesized cyanotoxins in the anaerobic environment that existed at that time, but they also developed methods to circumvent this toxicity. Both HCN and cyanate are toxic to the aerobic forms of life that evolved during the GOE (Stark, 1965) and cyanase was one enzyme capable of neutralizing the effects of cyanate. Thus, toxicity was likely a key impetus for the evolution or acquisition of cyanase among earth's early inhabitants but became less of a risk factor in more recently evolved, higher eukaryotes. and thus, gives cause to its absence in this population of animals This has been observed among the Nematoda however, to date no crown Clade V organisms have been identified that harbor a functional cyanase gene.

Plants also evolved defense mechanisms to protect themselves against invading pathogens and phytophagous arthropods. This entailed producing toxic cyanogenic glucosides that are hydrolyzed to HCN when chewed or digested and subsequently oxidized to cyanate (Wybouw et al., 2016). Most extant plants use β-cyanoalanine synthase to accommodate environmental and self-derived cyanide toxicity which involves interactions with Cox 1. but cyanase was acquired or evolved to address cyanate toxicity. If indeed early ancestors of *Trichinella* had an association with members of the plant kingdom, that association may have been hindered if its ancestors had not acquired a method to detoxify plant toxins. Ancestors of the plant root-knot nematodes *Meloidogyne* also acquired cyanase via HGT; but unlike ancestors of *Trichinella* this cyanase is more like those of bacterial origins (Haegeman et al., 2011; Jones et al., 2005; Mitreva et al., 2009). This anomaly i.e., plant parasitic nematodes with bacteria-derived genes, likely reflects an evolutionary change in trophism relative to early ancestors of *Meloidogyne;* akin to what is proposed for *Trichinella* spp. Phytophagous arthropods have evolved similar mechanisms to circumvent physical and biochemical barriers to herbivory including the breakdown of toxic plant-derived cyanogenic glucosides and the acquisition of bacteria-derived genes (Wybouw et al., 2016; Schoonhoven et al., 2005). Thus, there is ample support for a detoxification function for cyanase in the free-living ancestors of *Trichinella.*

Sung and Fuchs (1988) defined a *cyn* operon in *Escherichia coli* K12 consisting of a cyanate permease gene (T), the cyanase gene (S) and what was later determined to be a positive regulator of the LysR family (X). In recent years, genes for a carbonic anhydrase and a cyanate transporter, none of which have been linked to the *Trichinella* cyanase cassette, have also been associated with cyanase activity in bacteria (Fig. 5). However, even among different lineages of bacteria, the presence of the *E. coli cyn*TSX operon is not conserved. Members of the freshwater cyanobacterium *Synechococcus* possess a *cyn*ABDS gene cluster that requires a CO_2_ -concentrating mechanism and light for activation, as well as an ABC-type cyanate transporter. Species of *Pseudomonas* and proteobacteria possess a *cyn*FABDS that includes a transcriptional regulator CynF. These data are consistent with substantial evolution in the operation of the *cyn* gene cluster (Espie et al., 2007; Sáez et al., 2019). The absence of a cyanate transporter among the Clade I nematodes also indicates that the acquisition of cyanase may have been for the sole purpose of detoxification which would not account for cyanate as an energy source. Today, however, following its transition to parasitism, cyanase may have been co-opted by *Trichinella* for other than detoxification purposes and is now used as a source of recurring energy in an otherwise dormant ML.

### 3.2 Energy metabolism and nutrient utilization of cyanate

While the data support some relationship between ancestors of *Trichinella* and the plant Kingdom or symbionts thereof, hypotheses for the role of cyanase in extant *Trichinella* beyond detoxification are more difficult to ascertain but may be gleaned by examining alternative uses of the enzyme in other systems. Even though cyanate exists in nature in low levels (Mooshammer et al., 2021), many extant organisms exhibit a greater proclivity to utilize cyanate as an energy source than process it as a toxic substrate. Understandably, this is observed more so in single celled organisms (Mooshammer et al., 2021).

Bacterial cyanases have been linked to a multitude of physiological attributes one of which is the generation of alternative energy sources via the assimilation of nitrogen and NH_3,_ and the extraction of carbon from CO_2_ (Kitzinger et al., 2019; Luque-Almagro et al., 2008; Pachiadaki et al., 2017; Palatinszky et al., 2015). Anderson et al. (1990) found that *E. coli* could grow in as much as 20 mM cyanate; but that cyanase alone was insufficient to overcome the toxicity of cyanate which required permease activity as well. They concluded that the *cyn* operon functioned in both detoxification and energy metabolism. Later, Kozliak et al. (1995) proposed that in extant *E. coli,* cyanase-catalyzed decomposition of cyanate as the sole source of nitrogen was likely the primary function of cyanase rather than detoxification.

Brown and Wernegreen (2019) made a rather unique discovery when using clusters of orthologous groups to evaluate HGT genes in the in gut-associated Acetobacteraceae. Their results showed stark inconsistencies across functional categories, but that genes linked to energy production and conversion were among those with the greatest acquisition frequency in HGT. They also found that 15% of those genes were transporters; a subset of genes most likely to engender immediate advantage to a cell following transfer. This study corroborated work by Fuchsman et al. (2017) who found in bacteria and archaea, that HGT was highest among organisms sharing anaerobic and/or high temperature environments and that acquisition was over-represented by genes associated with energy conversion.

**Fig. 6:**
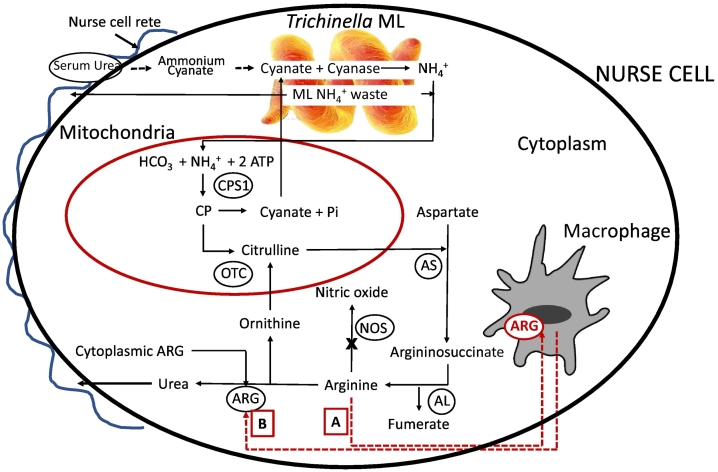
Hypothetical sources of cellular cyanate with access to *T. spiralis*. Potential sources of cyanate are designated by dashed lines as are mechanisms for [A] depleting cellular arginine via nutrient flux into macrophages or [B] liberating macrophage-derived arginase into the cell cytoplasm to control arginine concentration. Mechanisms are based upon a pseudo-urea cycle which has been demonstrated in muscle cells. Enzymes facilitating such a cycle are designated as CPS1 = Carbamoyl Phosphate Synthetase 1; OTC = Ornithine Transcarbamylase; AS = Argininosuccinate Synthetase; AL = Argininosuccinate Lyase; NOS = Nitric Oxide Synthase; ARG = Arginase 1. Pi = inorganic phosphate. The rete is presumed to envelop the entire NC.

Cyanate as the sole energy source was also identified among nitrifying bacteria. Palatinszky et al. (2015) showed that the nitrifier *Nitrososphaera gargensis* whose cyanase gene clusters with the genus *Nitrospira* (Fig. 2) could grow in an environment where cyanate only was used as the energy source. Like *Trichinella* and the other Clade I nematodes, neither a cyanate nor a nitrite transporter was detected within the *cyn* cassette of *Nitrososphaera.* The investigators concluded that transport of cyanate was likely not required and suggested that the flux of cyanate was facilitated by diffusion through the cell membrane from the surrounding ecosystem. In like manner, cyanate as a breakdown product of serum urea or internally generated carbamoyl phosphate (CP) (Fig. 6), may also not require transport in *Trichinella* and thus, give cause to the absence of such a transporter. Future work might evaluate the presence of cyanase in the cytoplasm of the NC and in the ES products of the ML. Its presence within in the NC could alter our perspective on assimilating cyanate as an energy course and direct functionality toward controlling cell toxicity; however, the absence of any of the 5 major signal peptides (Teufel et al., 2022) in *Trichinella* cyanase largely precludes the secretion of the cyanase into the NC.

Unfortunately, less research has been directed at the role cyanase plays in eukaryotes. Elleuche and Pöggeler (2008) were among the first to characterize a eukaryotic cyanase. The recombinant gene product was capable of degrading cyanate; however, research led the authors to conclude that the function of cyanase in *Sordaria macrospora* was ultimately not related to detoxification since growth inhibition by excess cyanate was revered in the presence of exogenous arginine. This afforded a link between the presence of cyanate and the growth of the fungi.

## 4 Sources of host-derived cyanate

Given the current ecological niche occupied by *Trichinella* and the timeline proposed for HGT, it is difficult to envision a present condition where controlling cyanate toxicity in host tissues is a primary function of the *Trichinella* cyanase. Further, elevated transcription of the cyanase gene in the larval stages supports a condition where cyanate is not environmentally derived. Whether one ascribes to a theory linking the *Trichinella* cyanase to detoxification or to assimilation as an energy source, identifying the source of host-derived cyanate utilized by or detoxified by extant organisms may assist in establishing its current function. Attempting to ascertain cyanate production in a muscle cell perturbed by the presence of a *Trichinella* larva complicates matters further. Several plausible sources of cyanate exist and can be either cellular or systemic in nature. To this end, understanding key pathways in host cells and *Trichinella* may provide insights into the production and availability of cyanate and the ultimate purpose for the *Trichinella* cyanase in the tissues of infected organisms. We will discuss below how CP and serum urea can be sources of host-derived cyanate, and how infiltrating anti-inflammatory type macrophages can facilitate the production of cyanate by helping to control arginase I levels inside the NC.

### 4.1 Carbamoyl phosphate

CP exists in nearly all living organisms and has been linked to prebiotic earth and the synthesis of pyrimidines, arginine, and the conversion of ADP to ATP (Jones, 1963; Shi et al., 2018). The importance of CP in key biosynthetic pathways cannot be overemphasized. Walsh et al. (2018), noted that CP is among the top eight most influential cellular components that use group transfer chemistry to power cell metabolism. These authors rank CP along with ATP, O_2_, NADPH and acetyl-CoA in importance. Today, CP functions anabolically as an intermediate in the synthesis of pyrimidines and arginine, and catabolically in the synthesis of urea. CP can be generated in vivo by multiple pathways. The predominant pathway involves mitochondrial-derived CP synthetase I (CPS I) which catalyzes the synthesis of CP from ammonia and has up to 1000-fold greater activity that CPS II which is used in the synthesis of pyrimidines and found in the cytosol; however, alternative mechanisms for CP biosynthesis have been observed in some bacteria that involve carbamate kinase or catabolic ornithine transcarbamylases. Given that CPS I is the key producer of CP in mammalian cells, it exudes extreme control over the levels of cellular ammonia. Most importantly, since cyanate is a natural decomposition product of CP (Allen and Jones, 1964; Suzuki et al., 1996), disrupting pathways that utilize CP can be a source of toxic cyanate or energy laden cyanate for the *Trichinella* ML (Fig. 6).

Is there a link between CP and *Trichinella*? Prior data showed that transcription of the *Trichinella* cyanase is highest in the L1, both the newborn larvae (NBL) and ML, possibly in preparation for muscle cell penetration and encystment (Zarlenga et al., 2019). To a lesser extent, cyanase gene expression was also observed in adult worms (Zarlenga et al., 2019), but it is unclear if this expression is adult worm derived or from in utero NBL. Adult worms do penetrate the columnar epithelium of the small intestine, but the infection results in a path of cell destruction rather than one of cell residency like the ML (Ren et al., 2011). Thus, tissue migration in the intestines lacks features of NC habitation and therefore would not exclude adaptive importance of cyanase to the muscle phase. It is also not known if the protein is produced following natural digestion of the NC and release of the ML in the intestinal tract and development to L5 in the intestinal epithelial cell. If so, this may pose a more vulnerable target for control of infection in the host. It should be noted that among the 36 other nematodes within which cyanase genes have been identified, nearly all have at least one tissue-dwelling stage. Thus, the current data suggest that *Trichinella*, CP and cyanase are linked by association to the NC, a relationship that is supported by examining the decomposition products of CP and control over CPS I. At physiological pH and temperature, CP spontaneously breaks down to cyanate, a relatively unstable molecule with a rather short half-life (t_1/2_ = 5 min). Given that *Trichinella* lacks pathways to synthesize urea, pyrimidines, arginine, and arginase 1, and lacks the genes to produce functional CPS I or CPS II enzymes, the parasite must rely on the host cell for one or more of these substrates and likely imparts control over the NC during and after development to accomplish this task. A vicious cycle can ensue if there is a deficiency in the supply of cellular ornithine where CP will accumulate and cause an increase in ammonia. This will provide more substrate for host CPS I which will generate more CP that will either decompose to cyanate or spill over to the cytoplasm for pyrimidine biosynthesis (Fig. 6). The cyanate can then be taken up by the worm and converted to ammonia thereby renewing a mini cycle.

In bacteria, (Anderson et al. (1973) demonstrated that the activity of CPS is reduced in the presence of elevated levels of cyanate. This results in a concomitant decrease in cell growth by decreasing the production of citrulline from ornithine. Because growth inhibition is reversed by the addition of exogenous arginine, Guilloton and Karst (1987) concluded that any shortage in CP had a greater impact on arginine biosynthesis than on the synthesis of pyrimidines. Relating these findings to the niche that *Trichinella* has created for itself in the NC, it is possible that the worm commands a delicate balance in the levels of host-derived cyanate, CP, CPS, arginine and arginase 1 to control cell growth and development without killing the NC. This is exemplified in part by muscle cell growth becoming permanently suspended in the G2/M phase after infection. Also, evidence of elevated levels of ribosomal RNA in the NC suggests a concomitant increase in biosynthesis while the NC is in a suspended state of development.

The early phase of the muscle infection is associated with the acquisition of the NC phenotype and exponential growth of the ML. This is followed by the second phase or maintenance of the NC phenotype and maturation of the ML where each phase exhibits distinct needs. In the early phase of NC development, there is a demonstrable increase in the number and size of euchromatic nuclei (Despommier et al., 1991). With this, one can hypothesize a concomitant increase in pyrimidine biosynthesis which in turn would proportionally elevate CP production. The resulting cyanate from unutilized CP may sustain the infecting worm during the initial growth spurt but may not support the worm long term when 1) transcriptional activation of muscle specific genes become curtailed, 2) the cell arrests in the G2/M phase of development and 3) a new infected cell phenotype emerges (Jasmer, 1993). At this time, the worm's excretory secretory (ES) products may elicit continuing CP production. This hypothesis is supported in part because in addition to the heavily studied immunodominant ES antigens secreted by *Trichinella* ML, ES products are also comprised of amines where ammonia accounts for 30–50% of excreted nitrogen. Indeed, 100,000 ML will secrete 400–600 μg of nitrogen over a 24 h period (Haskins and Weinstein, 1957). Clearly, quantitative measurements on isolated ML may not reflect the true biology of encysted ML; however, the absence of a urea cycle does implicate the secretions of ammonia and amine byproducts by encysted ML. Excess ammonia released by the encysted ML may provide adequate substrate for the continuing production of CP by CPS from which cyanate will be generated if not used by the cell. Normally, a cell has feedback mechanisms to regulate substrate immoderations; however, given the NC is a metabolically perturbed system, the biochemistry and physiology of the cell may be largely controlled by the worm. Alternatively, the parasite may exert influence over the cell whose own altered genetic programing may determine the foundation upon which the parasite can work. One outcome of uncontrolled high levels of cyanate would be untethered carbamylation of cell proteins and the demise of the NC This hypothesis supports the utilization of cyanase in the creation of an energy efficient niche for worm longevity rather than only as a detoxifying pathway; however, as a source of energy, the catabolism of cyanate would suffice for both.

### 4.2 Urea

Many studies on *Trichinella* have focused on immunity and host:parasite relationships; less effort has been afforded to the biochemistry of the infection. Herein may rest testable hypotheses for the current purpose of the *Trichinella* cyanase. Generally, nematodes including *Trichinella* spp. lack the archetypal metazoan ornithine-urea cycle; eukaryotic algae and plants also lack a prototypical urea cycle (Horák et al., 2020). Rather than convert ammonia to urea, *Trichinella* spp. secrete unprocessed nitrogen containing compounds in their ES products which may help control host metabolism, the use of arginine, arginase I and associated pathways, and provide a common thread in the health of *Trichinella* ML and the catabolism of cyanate. Elleuche and Pöggeler (2008) noted in filamentous fungi, that elevated arginine levels down-regulate cyanase activity suggesting that cyanase functionality is optimal under conditions of arginine deficiency; a predictable outcome of elevated arginase I levels. This gives cause to the elevated levels of arginase I enzyme in the NCs and infiltrating immune cells.

Cyanate exists in equilibrium with urea where approximately 0.8% of serum urea naturally decomposes to ammonium cyanate (Dirnhuber and Schütz, 1948) (Fig. 6). This raises the possibility that the cyanate used by/degraded by *Trichinella* is derived from the breakdown of serum urea. Humes and Akers (1952) and later Despommier (1998) showed at full development, that the NC is enveloped by a vascular network from which the worm is postulated to extract nutrients as part of its survival process. This makes serum a potential source of cyanate for the encysted worm. However, Pardridge et al. (1982) demonstrated in culture, that rat striated muscle cells derived from regenerating hindlimbs were well capable of synthesizing ornithine and urea, and that production was linked to the utilization of serine and arginine in the presence of high levels of cell-derived arginase I which has been linked to a multitude of human diseases (Caldwell et al., 2018). Pandya et al. (2019) also reported elevated levels of arginase I in aged muscle cells as did Wu et al. (2001) who linked arginase I levels in rat aortic smooth muscle cells to the escalation of cell proliferation. Thus, all forms of muscle cells are capable of producing urea from which cyanate may arise and generating arginase I as an indispensable enzyme in the urea cycle.

Some have likened the reprogramming of the NC to a regeneration process following cell injury, and to wound healing and muscle cell repair on a micro rather than macro scale (Wu et al., 2008) The newborn larvae use stylets to actively pierce or damage the muscle cell prior to penetration thereby inducing mechanical damage to the cell early in the infection process. Albina et al. (1988) found elevated levels of arginase activity and ornithine in wounded tissues possibly derived from infiltrating macrophages. Using a mouse model, Campbell et al. (2013) reported that arginase I is actively regulated during wound healing, and they linked a delay in healing to a down regulation of arginase. Though this may originate from infiltrating M2-type macrophages, damaged cells typically initiate the healing process by increasing production of arginine and ornithine which in turn increases proline production which is important in collagen synthesis. The polyamines i.e., spermidine and spermine, which are actively secreted by the ML (Hamana et al., 2006) are also important in the production of collagen and may play a role in capsule formation. Cyanate coming from the vascular network through the NC membrane and into the cytoplasm may therefore function collaboratively with Ts-secreted polyamines to generate the collagen capsule.

### 4.3 Immune cells

Host immune responses to *Trichinella* infection are quite complex given the nature of the infection, the distinct niches occupied by adult worms and ML, and variations in the biochemistry of these stages. While immunologically complex, there may be additional factors associated with the infiltration of immune-related cells in and around the NC indirectly related to immunity. Macrophage cells have been observed in the cellular infiltrates that surround the NC (Beiting et al., 2004; Karmańska et al., 1997a). Of equal and possibly greater importance, macrophages have also been observed within the NC (Karmańska et al., 1997b). In general, macrophages are polarized to M1 (pro-inflammatory) or M2 (anti-inflammatory) metabolic phenotypes where the cognate arginases are important in the production of nitric oxide (NO) and citrulline, or urea and ornithine, respectively. Excess arginase I can also down regulate NO production by commandeering available arginine needed to advance the urea cycle. Furthermore, the M2-type participates in generating a Th2 immune response, and in promoting tissue repair. The Th-2 response is part of the adaptive immune system, specifically, acquired immunity, and is involved in activating antibody-mediated, immune responses against extracellular pathogens including parasites. Data indicate that macrophages in and around the NC are polarized to the M2-type. Okada et al. (2013) showed that *Trichinella* infections significantly increased the M2/M1 ratio in mouse macrophages and later it was shown that the 53 kDa ES antigen from *T. spiralis* can polarize peritoneal macrophages (Du et al., 2014) mouse lung macrophages (Wei et al., 2021) and macrophages in endotoxemic mice (Chen et al., 2016) to the M2-type.

Over the years, it has become evident that the metabolism of arginine by immune cells is fundamentally involved in a plethora of diseases and infections. Thus, a key link to these findings and to the production of cyanate is that the M2-type macrophage synthesizes arginase I which is instrumental in urea and ornithine production. Also, in the presence of infiltrating eosinophils, Fabre et al. (2009) did not observe NO II in the area surrounding the NC which agrees with M2 polarization where the arginase 1 limits the availability of arginine for NO production (Gotoh and Mori, 1999). Thus, the biochemistry of infiltrating macrophages may actually help engender a biological niche for *Trichinella* that is high in cyanate by depleting local arginine concentrations via the flux of nutrients into surrounding or NC-derived macrophages (Chen et al., 2022; Munder et al., 2009; Pesce et al., 2009; Rodriguez et al., 2010) or by liberating arginase-1 into the adjacent environment or directly into the NC (Davis et al., 2021; Munder et al., 2006) which in turn would cause an elevation in CP production (Fig. 6). Although immune cell interactions are most evident early in the infection, later inflammatory cell infiltration can drop off markedly, yet the parasite remains viable. Thus, any involvement of macrophages in the life of the NC may be more applicable during the early phases of establishment. It should be noted that any participation by immune cells does not preclude the acquisition of similar nutrients and substrates via the NC rete (Fig. 6).

## 5 Summary

Horizontal gene transfer resulting in new metabolic pathways for cyanase among nematodes occurred minimally through 2–3 independent events in deep evolutionary time. We infer independence of these events phylogenetically, based on the putative sources for pathways derived from plants (and their symbionts) in some basal nematodes (among Clade I = Enoplea, containing Subclass Dorylaimia) or from proteobacteria in crown taxa (among Clade III = Rhabditida, and Clade IV = Rhabdititda and Tylenchida), and from the common ancestor for these latter lineages (Clade designations according to Blaxter et al., 1998; Holterman et al., 2006; Meldal et al., 2007 with classification from Hodda, 2007).

Given that mammals do not themselves possess a cyanase gene, the reason for the contemporary function of this pathway among a limited diversity of Dorylaimea, particularly the assemblage of parasitic genera and species of the Trichocephalia remains an enigma. Deep evolutionary, free-living ancestors of *Trichinella,* would have acquired this gene via HGT. However, representatives of contemporary trichinellid taxa, and species of *Trichinella* retained this gene product as part of their protein repertoire. Even though mammals do not have a problem with cyanate and its toxic effects, we believe that cyanase is nonetheless an integral part of the *Trichinella* life cycle within the host. Although the host cell infected with *Trichinella* survives, its character has been so severely perturbed that it is no longer referred to as a muscle cell but a nurse cell. Thus, it is assumed that biochemical pathways that support normal muscle cell growth and development have been equally perturbed. With this mindset there exist multiple scenarios by which cyanate can be generated and/or accumulate in the NC if the conditions are suitable, in particular, via pathways related to the urea cycle and pyrimidine biosynthesis.

Incontrovertible evidence has been presented that 1) the cyanase in *Trichinella* is functional, 2) the gene sequence clusters with genes from the Kingdom Plantae, 3) the gene was acquired basal to the Trichocephalia lineage before the radiation leading to the Trichinellida and Dioctophymatida, and 4) at least 36 other nematodes have also acquired functional cyanase genes by HGT through one or more independent events from proteobacteria (Zarlenga et al., 2019). Clearly the most challenging part of this phenomenon is that clustering of this gene with those of the Kingdom Plantae establishes a prior relationship between this group of eukaryotes and deep ancestors of *Trichinella*. Precedence has been advanced even within nitrifying bacteria that such a relationship is possible specifically as it relates to the acquisition of cyanase. We have presented plausible hypotheses for the function of cyanase in *Trichinella*, but it is yet unknown if activity is related to detoxification, assimilation as a source of energy, or a combination of both. Nonetheless, if one ascribes to the premise that a gene acquired by HGT must integrate into the recipient biology, advancing longevity and evolutionary persistence, then the *Trichinella* cyanase gene and/or gene product become viable targets for future approaches to attenuate the infection.

The following are the supplementary data related to this article.

## Declaration of Competing Interest

The authors declare that they have no known competing financial interests or personal relationships that could have appeared to influence the work reported in this paper.
